# Whole-genome sequencing of clarithromycin resistant *Helicobacter pylori* characterizes unidentified variants of multidrug resistant efflux pump genes

**DOI:** 10.1186/1757-4749-6-27

**Published:** 2014-06-26

**Authors:** Akira Iwamoto, Toshihito Tanahashi, Rina Okada, Yukio Yoshida, Kaoru Kikuchi, Yoshihide Keida, Yoshiki Murakami, Lin Yang, Koji Yamamoto, Shin Nishiumi, Masaru Yoshida, Takeshi Azuma

**Affiliations:** 1 Department of Internal Medicine, Division of Gastroenterology, Kobe University Graduate School of Medicine, 7-5-1 Kusunoki-cho, Chuo-ku, Kobe 650-0017, Japan; 2 Department of Medical Pharmaceutics, Kobe Pharmaceutical University, 4-19-1 Motoyama-kita, Higashinada-ku, Kobe 658-8558, Japan; 3 Division of Internal Medicine, Okinawa Prefectural Chubu Hospital, 281 Miyazato, Uruma-city, Okinawa 904-2293, Japan; 4 Department of Hepatology, Osaka City University Graduate School of Medicine, 1-4-3 Asahimachi, Abeno-ku, Osaka 545-8585, Japan

**Keywords:** Whole-genome sequencing, *Helicobacter pylori*, Clarithromycin, Multidrug efflux, TolC homolog

## Abstract

**Background:**

Clarithromycin (CLR) is the key drug in eradication therapy of *Helicobacter pylori* (*H. pylori*) infection, and widespread use of CLR has led to an increase in primary CLR-resistant *H. pylori*. The known mechanism of CLR resistance has been established in A2146G and A2147G mutations in the 23S rRNA gene, but evidence of the involvement of other genetic mechanisms is lacking. Using the MiSeq platform, whole-genome sequencing of the 19 clinical strains and the reference strain ATCC26695 was performed to identify single nucleotide variants (SNVs) of multi-drug resistant efflux pump genes in the CLR-resistant phenotype.

**Results:**

Based on sequencing data of ATCC26695, over one million sequencing reads with over 50-fold coverage were sufficient to detect SNVs, but not indels in the bacterial genome. Sequencing reads of the clinical isolates ranged from 1.82 to 10.8 million, and average coverage ranged from 90.9- to 686.3-fold, which were acceptable criteria for detecting SNVs. Utilizing the conventional approach of allele-specific PCR, point mutations in the 23S rRNA gene were detected in 12 clinical resistant isolates, but not in 7 clinical susceptible isolates. All sequencing reads of CLR-resistant strains had a G mutation in an identical position of the 23S rRNA gene. In addition, genetic variants of four gene clusters (*hp0605*-*hp0607*, *hp0971*-*hp0969*, *hp1327*-*hp1329*, and *hp1489*-*hp1487*) of TolC homologues, which have been implicated in multi-drug resistance, were examined. Specific SNVs were dominantly found in resistant strains.

**Conclusions:**

Gene clusters of TolC homologues are involved in CLR susceptibility profiles in individual *H. pylori* strains. Whole-genome sequencing has yielded novel understanding of genotype-phenotype relationships.

## Background

*Helicobacter pylori* (*H. pylori*) infection is recognized in approximately 50% of the adult population and associated with a wide range of upper gastrointestinal diseases [1,2]. Present treatments for *H. pylori* infection consist of a proton pump inhibitor based triple therapy in combination with amoxicillin and clarithromycin (CLR) [2]. Triple therapy has remained the first-choice regimen for the past decade and has been recommended by most consensus meetings, and by both European and American scientific societies [3-6].

In many cases, CLR is the key component of eradication therapy, but antibiotic resistance is the primary failure of triple therapy in patients with *H. pylori* infection. Prevalence of CLR-resistant *H. pylori* has become increasingly common in many countries [7,8]. Consequently, it has been suggested that the efficacy of standard triple therapy has been gradually reduced to unacceptable levels [9]. It is accepted that triple therapy should not be used to manage *H. pylori* infection when the prevalence of CLR resistance is higher than 15–20% [3]; in such cases, other therapeutic alternatives should be considered.

CLR binds to the peptidyl transferase region of the bacterial 23S rRNA and inhibits protein synthesis [10]. Acquired resistance of CLR in *H. pylori* has been associated with point mutations in the peptidyl transferase region of domain V of the 23S rRNA. Two copies of the 23S rRNA gene are present in *H. pylori*, and the most common mutation is an A-to-G transition at position 2146 and 2147 (previously known as A2142G and A2143G) [11]. Recent reports have also indicated that other 23S rRNA gene mutations might be associated with CLR resistance [12,13]. In addition, CLR resistance seems to be related with other factors, such as rRNA methylase production [14], the actions of macrolide-inactivating enzymes, and active efflux pumps, which have been described in several bacterial species [15]. Indeed, an active efflux pump has been shown to contribute to CLR resistance in *H. pylori*[16]. As a result of the worldwide increased rate of CLR resistance, some studies have indicated that other bacterial genetic factors might contribute to this increased resistance [17,18]. However, there is no genetic evidence for other bacterial factors in *H. pylori*.

To characterize the bacterial pathogen samples, recent advances in whole-genome sequencing, such as the development of DNA-sequencing technologies, were applied to this study [19]. Compared to conventional methods (e.g., capillary sequencing and non-sequence-based molecular methods), next-generation sequencing provides deep views on bacterial genome information without biases in downstream analysis [20]. These techniques are the ideal tool to comprehensively track extensive genomic knowledge of antimicrobial resistance and to facilitate specific and rational treatment of infected patients [21]. However, despite the broad advantages, next-generation sequencing requires the extensive bioinformatics tasks for data analysis from assembly to gene annotation, and this remains a significant problem for the routine application to clinical microbiology [22].

In this study, we first applied the established method of allele-specific PCR to detect 23S rRNA gene mutations in clinical *H. pylori* strains and identified CLR-resistant isolates. To elucidate the unidentified genetic factors in the reduced susceptibility to CLR in *H. pylori*, whole-genome sequencing was applied to 20 selected *H. pylori* strains, including the major laboratory strain ATCC26695, 12 resistant and 7 susceptible strains. Of note, we focused on variants of multidrug resistance (MDR) efflux pumps that contribute to both intrinsic and acquired antibiotic resistance and assessed their association with novel mechanisms of CLR resistance.

## Results

### AS-PCR for A2146G and A2147G mutations in 23S rRNA

**Figure 1:**
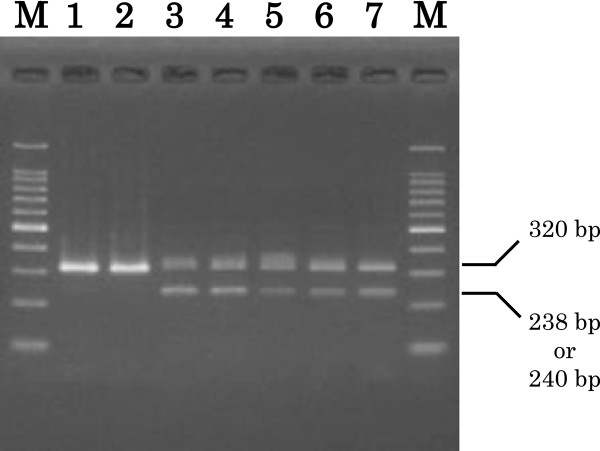
**Representative electrophoresis patterns of allele-specific PCR products.** PCR products were amplified with mixed primers for determination of A2146G and A2147G mutations in 23S rRNA of *H. pylori*. PCR products of 320 bp are commonly observed for the *H. pylori* 23S rRNA gene. PCR products of 238 or 240 bp indicate the presence of a point mutation in the 23S rRNA gene. PCR products were obtained from ATCC26695 (lane 1), J99 (lane 2), and five representative CLR-resistant strains (lanes 3–7; S1, S13, S17, S26, and 174). The 100 bp marker ladder is indicated by M.

For 19 clinical isolates, AS-PCR clearly differentiated the 23S rRNA mutants from the *H. pylori* (Figure 1). Point mutations in the 23S rRNA gene at position 2146 or 2147 were detected in 12 strains and defined as CLR-resistant isolates in this study. Twelve mutant strains were isolated from patients who failed eradication therapy, and 7 wild-type strains were isolated from patients with successful eradication therapy. Thus, AS-PCR results were in agreement with the outcome of eradication therapy based on clinical information (data not shown).

### ATCC26695 total reads and average coverage

Prior to analysis of clinical isolate sequencing data, we generated six independent sequencing data sets from ATCC26695 to determine the adequate total reads and average coverage that should be targeted for our study (Table 1). Illumina MiSeq generated 150-bp paired-end reads with 110,650 to 2,148,576 reads from the six samples. After trimming, the number of total reads was nearly unchanged before trimming. When all data were mapped to the theoretical nucleotide length of ATCC26695 (1,667,867 bp) using Genomics Workbench 6.0.1, the total consensus coverage (%) ranged from 87.38 to 99.99%, and average coverage was from 4.2- to 81.7-fold. In the sequencing data from sample 5 and 6, each with over one million reads, we found nearly equal total consensus coverage (99.98 and 99.99%, respectively) and average coverage (71.8- and 81.7-fold, respectively).

**Table 1:** Six ATCC26695 sequence data sets

**Sample ID**	**Total reads (before trimming)**	**Total reads (after trimming)**	**Total consensus length (bp)**	**Total consensus coverage (%)**	**Average coverage (fold)**	**Assemble gaps (bp)**	**NCBI accession number**
1	110,650	109,952	1,457,400	87.38	4.2	210,467	NA
2	130,226	130,216	1,649,483	98.89	5.9	18,384	NA
3	488,824	488,724	1,667,405	99.97	29.5	462	NA
4	664,278	642,532	1,667,519	99.97	36.3	348	NA
5	1,211,740	1,195,756	1,667,544	99.98	71.8	323	NA
6	2,148,576	2,107,718	1,667,758	99.99	81.7	211	DRS013117

Total reads after trimming are mapped to the reference nucleotides of 1,667,867 bp (NC_000915) and calculated with Genomic Workbench 6.0.1. NA; not available.

### Quality control of ATCC26695 sequencing data

Adapter sequences were automatically trimmed from the reads with MiSeq reporter analysis. As shown in Table 1, the sequencing data from sample 6 of ATCC26695 was subjected to the quality control analysis.

As a measure of quality control, we determined the length distribution of sequencing reads, ambiguous base-content, and quality distribution at each base position (Additional file 1: Figure S1). This analysis led to the identification and subsequent removal of several errors in the raw sequencing data. After trimming, histograms of sequencing lengths distribution shifted to a shorter sequence length, and there was no ambiguous base-content. The mean values of Phred scores were >30 at all base positions up to 150 bp. Thus, quality control assessment showed that the entire length from the 150-bp paired-end reads could be analyzed. The FASTQ file, containing the trimmed sequences, was used for further analysis.

### Coding sequence from ATCC26695 sequencing data

ATCC26695 sample 6 was chosen for further analysis based on its total number of reads, read quality, and coverage. Sequencing reads were aligned to the NC_000915 genome using Genomic Workbench 6.0.1. Analytical procedure was shown in Additional file 2: Figure S2 as a flow chart.

Despite over two million sequencing reads of ATCC26695, a total of 211 bp was identified as an assembly gap to the NC_000915 reference sequence (Table 2). The detailed assemble gaps were as follows; a total of 51 bp in 48 short insertions, 27 bp in 25 short deletions, and 133 bp in 4 long deletions. The quality and coverage of the sequence data was effective to determine the differences between isolates of a single laboratory strain. In our sequencing data, seven nucleotides per genome (1.66 Mb) were different from the reference nucleotides with over 50% frequency (Table 3).

**Table 2:** Assemble gaps of ATCC26695 sequence data from sample 6

**Type**	**Length (bp)**	**Sequence**		**Nucleotide position in reference sequence**		**Locus tag**	**Locus_tag (5' ? 3')**	**Annotation**	**Coverage**
		**Insertion**	**Deletion**	**Start**	**End**				**Insertion: consensus side/Deletion: reference side**
**Short insertion**									
1	1	A		39762	39763	HP0041	→	hypothetical protein	63
2	1	G		159581	159582	HP0147	→	cytochrome c oxidase, diheme subunit, membrane-bound (fixP)	89
3	1	C		173256	173257	HP0165	←	hypothetical protein	94
4	1	G		208167	208168	HP0203	→	hypothetical protein	85
5	1	C		263286	263287	HP0253	→	hypothetical protein	105
6	1	C		331675	331676				2
7	1	T		331678	331679				2
8	1	G		331680	331681				2
9	1	T		331682	331683				2
10	1	G		474218	474219				88
11	1	C		475466	475467	HP0456	→	hypothetical protein	53
12	1	C		475645	475646				46
13	1	T		524751	524752	HP0498	→	sodium- and chloride-dependent transporter	54
14	1	G		579891	579892				76
15	1	A		626216	626217				57
16	1	A		674290	674291	HP0628	→	hypothetical protein	63
17	1	C		683744	683745	HP0636	←	hypothetical protein	54
18	1	G		739962	739963	HP0688	→	hypothetical protein	45
19	1	T		745341	745342	HP0694	→	hypothetical protein	84
20	1	T		745352	745353				86
21	1	A		745389	745390				95
22	1	T		745429	745430				97
23	2	AG		773423	773424	HP0719	→	hypothetical protein	55
24	1	C		787709	787710	HP0732	←	hypothetical protein	62
25	1	G		808800	808801	fliD	→	flagellar capping protein	57
26	1	C		838354	838355	HP0783	←	hypothetical protein	74
27	1	A		843491	843492				69
28	1	G		861042	861043	HP0807	←	iron(III) dicitrate transport protein (fecA)	86
29	1	G		888773	888774	HP0836	→	hypothetical protein	98
30	2	AG		915893	915894	HP0863	←	hypothetical protein	112
31	1	A		915897	915898	HP0863	←	hypothetical protein	112
32	1	C		993909	993910	HP0931	→	hypothetical protein	109
33	1	G		1026924	1026925				63
34	1	T		1057531	1057532				69
35	1	T		1057599	1057600				59
36	1	T		1077873	1077874	HP1014	→	7-alpha-hydroxysteroid dehydrogenase	83
37	1	G		1207116	1207117	HP1144	→	hypothetical protein	74
38	1	C		1207608	1207609	HPr04	←	16S ribosomal RNA	101
39	1	G		1208482	1208483	HPr04	←	16S ribosomal RNA	1
40	1	C		1208483	1208484	HPr04	←	16S ribosomal RNA	2
41	1	T		1256328	1256329	HP1186	→	carbonic anhydrase	72
42	1	C		1259151	1259152				113
43	1	A		1264196	1264197	HP1193	→	aldo/keto reductase	92
44	1	G		1291618	1291619	HP1216	←	hypothetical protein	74
45	2	GG		1475694	1475695	HPr06	←	23S ribosomal RNA	1
46	1	C		1511030	1511031				4
47	1	C		1511162	1511163	HPr04	←	16S ribosomal RNA	83
48	1	C		1511398	1511399	HPr04	←	16S ribosomal RNA	11
Total	51								
**Short deletion**									
1	1		A	25622					58
2	1		C	38695		HP0039m	→	conjugal plasmid transfer system protein	62
3	2		YC	129158	129159				1
4	1		C	342188		HP0326	→	CMP-N-acetylneuraminic acid synthetase	86
5	1		T	426389		HP0413	←	transposase-like protein, PS3IS	18
6	1		A	463967		HP0445	←	hypothetical protein	75
7	1		A	593944					81
8	1		G	650897		HP0609	→	hypothetical protein	73
9	1		A	674221		HP0627	→	hypothetical protein	47
10	1		T	728119		HP0678	←	hypothetical protein	75
11	1		G	745422					98
12	1		C	815405		HP0760	←	phosphodiesterase	41
13	1		C	815420		HP0760	←	phosphodiesterase	48
14	1		R	1027030					73
15	1		G	1049706					58
16	1		G	1071033		HP1009	←	site-specific recombinase	77
17	1		T	1077870		HP1014	→	7-alpha-hydroxysteroid dehydrogenase	87
18	1		G	1081560		HP1018	→	hypothetical protein	97
19	1		G	1192591		HP1127	←	hypothetical protein	61
20	1		T	1256382					81
21	1		C	1258321		HP1188	←	hypothetical protein	71
22	1		T	1264200		HP1193	→	aldo/keto reductase	91
23	2		NA	1480608	1480609	HP1410	→	hypothetical protein	1
24	1		A	1480611		HP1410	→	hypothetical protein	1
25	1		A	1480614		HP1410	→	hypothetical protein	1
Total	27								
**Long deletion**									
1	42		TGATTATATTTGTAATGGTGCTCRCTTGTTTAAAATGAGYCT	129001	129042	HP0118	←	hypothetical protein	not calculated
2	61		KTCTTTTGGGTTTTGTAAAAATTACCTCCTTAATTTGGTTTTGTTTTGTTTAGACTTTAAC	425976	426036	HP0413	←	transposase-like protein, PS3IS	not calculated
3	14		GGATGGGTGCTTTT	1475703	1475716	HPrrnB23S	←	transposase-like protein, PS3IS	not calculated
4	16		NATCCGCGCTTAAGCG	1480568	1480583	HP1410	→	hypothetical protein	not calculated
Total	133								

**Table 3:** SNVs of ATCC26695 sequence data from sample 6

	**Nucleotide position (NC_000915)**	**Reference nucleotide (NC_000915)**	**Mutated nucleotide**	**Total reads**	**Reads of mutated nucleotide**	**Frequency of mutated nucleotide (%)**	**Annotations**
1	130106	T	C	217	117	53.9	Gene: HP0119
2	130111	T	G	186	94	50.5	Gene: HP0119
3	301322	G	T	109	108	99.0	Gene: HP0289
4	1370263	G	C	101	99	98.0	Gene: rpmJ
5	1428910	C	A	137	104	75.9	Gene: HP1366
6	1502547	T	C	345	184	53.3	
7	1502549	A	G	345	184	53.3	

Total reads and mutated reads indicate the number of reads that passed quality control.

Taken together, these sequencing data indicated that over one million sequencing reads had >50-fold average coverage of the assembled contigs when reads were mapped to the reference *H. pylori* genome. Since it was difficult to identify true single-base and small indels during our mapping process, we did not try to identify any size of indels in further analysis of the clinical isolates.

### Whole-genome sequencing of 19 clinical *H. pylori* strains

All *H. pylori* strains were sequenced with illumina MiSeq. After triminng, total reads of 7 wild-type (susceptible) strains ranged from 1.96 million to 5.32 million (Table 4A). Similarly, total reads of 12 resistant strains ranged from 1.82 million to 10.8 million (Table 4B). After trimming above-mentioned, sequencing data were mapped to the ATCC26695 reference genome. Average coverage ranged from 90.9 to 283.6-fold in susceptibile strains and from 125.5 to 686.3-fold in resistant strains. These data indicated that the derived high sequencing depth of coverage was sufficient to detect high quality SNVs in the *H. pylori* genome.

**Table 4:** **Whole-genome sequencing of 19 clinical *H. pylori *** **isolates**

**Strain**	**Total reads (before trimming)**	**Total reads (after trimming)**	**Total consensus length (bp)**	**Total consensus coverage (%)**	**Average coverage (fold)**	**NCBI accession number**
**CLR-susceptible**						
177	2,619,844	2,563,352	1,666,155	99.89	116.3	DRS013118
179	2,291,578	2,269,154	1,560,395	93.55	131.8	DRS013119
189	2,010,838	1,967,720	1,664,913	99.82	90.9	DRS013120
F32	5,383,222	5,327,650	1,593,944	95.56	283.6	DRS013121
F44	3,887,082	3,806,902	1,667,084	99.95	157.9	DRS013122
F65	4,248,748	4,157,272	1,667,173	99.95	189.6	DRS013123
F79	3,036,516	3,035,946	1,562,308	93.67	190.1	DRS013124
**CLR-resistant**						
S1	2,271,944	2,271,890	1,571,448	94.21	149.2	DRS013125
S2	5,962,628	5,962,428	1,587,965	95.20	342.2	DRS013126
S4	4,640,490	4,640,330	1,578,524	94.64	262.8	DRS013127
S8	10,869,516	10,868,918	1,610,753	96.57	686.3	DRS013128
S13	3,873,394	3,873,000	1,563,976	93.77	264.2	DRS013129
S16	6,782,392	6,781,628	1,594,857	95.62	448.2	DRS013130
S17	5,700,038	5,699,336	1,596,480	95.71	327.3	DRS013131
S22	6,597,254	6,596,450	1,598,844	95.86	426.8	DRS013132
S23	1,820,652	1,820,436	1,559,100	93.47	125.5	DRS013133
S26	6,692,038	6,691,390	1,584,702	95.01	442.9	DRS013134
174	3,004,008	3,003,900	1,580,603	94.76	169.7	DRS013135
194	6,936,714	6,921,552	1,600,238	95.94	415.2	DRS013136

Total reads after trimming are mapped to the reference nucleotide of ATCC26695 and calculated with Genomic Workbench 6.0.1.

### Detection of A2146G and A2147G with sequencing reads

Prior to analyzing the target genes in the derived sequence data, we examined the position of point mutations in 23S rRNA to identify any false positive variants. Two identical copies of 23S rRNA (HPr01 and HPr06) are found in the *H. pylor*i genome [11].

**Figure 2:**
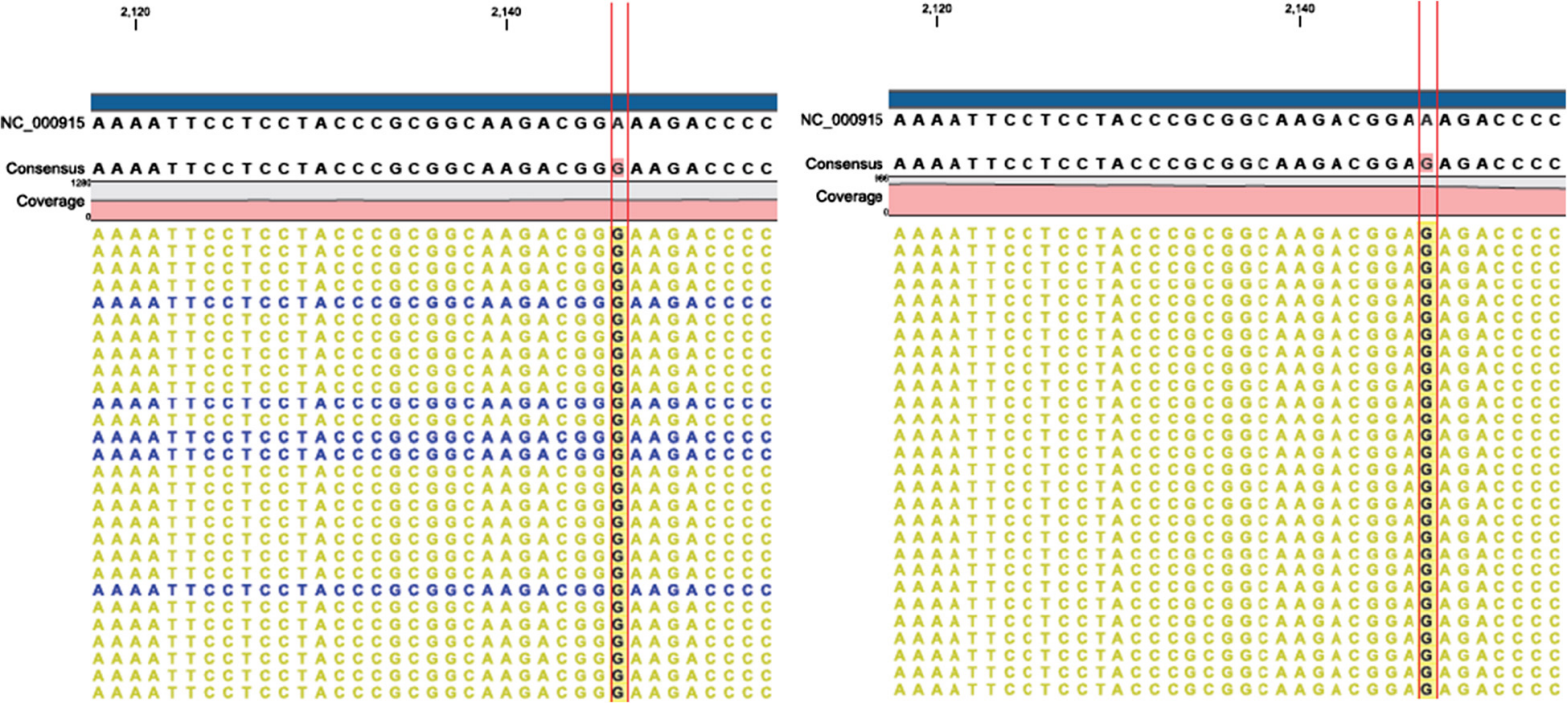
**A2146G and A2147G mutations in the 23S rRNA gene identified by illumina sequencing.** The aligned results of sequencing reads of two CLR-resistant *H. pylori* strains (S17 and S26) are shown. Along the top, reference nucleotide sequences of NC_000915 (ATCC26695), and consensus nucleotide sequences of each CLR-resistant strain are shown (black). The lower portion of the sequence viewer shows mapped sequencing reads (yellow and blue). In 479 reads from S17 strain (left), reference nucleotide A was changed to G at position 2146 in 23S rRNA (bold faces). Similarly, reference nucleotide A was changed to G at position 2147 in 23S rRNA in 485 reads from S26 (right).

Sequence data of 12 resistant strains mapped to the ATCC26695 reference genome. The results of two representative resistant strains, S17 and S26, are shown in Figure 2. In the S17 strain at position 2146 in the 23S rRNA gene, all sequence reads (479-fold) had the variant G. Similarly, in S26 at position 2147, all reads (485-fold) had the variant G. In the other copy of the 23S rRNA, the same changes were found in each strain. Sequencing reads of the remaining 10 strains also contained the variant G. The AS-PCR is a targeted approach for identifying variants at one base-pair position (i.e., 2146 or 2147), but whole-genome sequencing can simultaneously detect multiple base-pair variants with high confidence. In contrast, there was no variant in the same positions of 23S rRNA genes of 7 CLR-susceptible strains. Representative result of F79 strain was shown in Additional file 3: Figure S3.

### Coding sequence (CDS) variants in efflux pump genes

To identify other possible variants in these clinical isolates, we next examined genetic variants of four gene clusters of TolC homologues, which have been associated with multidrug resistance [23]. To the best of our knowledge, this is the first survey of *H. pylori* MDR efflux transporter variants. During the filtering of uncertain indels and the analysis of > 99.0% of mapped reads, we created a list of high-quality informative SNVs associated with CDS variants (Table 5).

**Table 5:** SNVs of multidrug resistant efflux pump genes and MICs of CLR

**Strain**	**MICs of CLR (μg/ml)**	***hp0605 (hefA )***	***hp0606 (hefB )***	***hp0607 (hefC )***	***hp0969 (hefF )***	***hp0970 (hefE )***	***hp097 *** **1 *(hefD )***	***hp1327 (hefG )***	***hp1328 (hefH )***	***hp1329 (hefI )***	***hp1487***	***hp1488***	***hp1489***	**Total**
**CLR-susceptible**														
177	-	0	0	0	0	0	0	0	0	0	0	0	0	0
179	-	29	12	70	51	13	3	22	16	47	30	26	31	350
189	-	0	0	0	0	0	0	0	0	0	0	0	0	0
F32	-	48	20	76	83	24	27	0	0	0	0	0	0	278
F44	-	0	0	0	0	0	0	0	0	0	0	0	0	0
F65	-	0	0	0	0	0	0	0	0	0	0	0	0	0
F79	-	39	15	49	55	16	30	0	0	0	0	0	0	204
**CLR-resistant**														
S1	32	32	17	51	55	9	25	23	18	61	23	13	27	354
S2	32	50	18	74	0	0	0	0	0	0	0	0	0	142
S4	32	39	18	67	55	16	26	0	0	0	0	0	0	221
S8	4	44	19	67	0	0	0	0	0	0	0	0	0	130
S13	16	52	21	80	76	22	31	0	0	0	0	0	0	282
S16	16	54	23	81	0	0	0	0	0	0	0	0	0	158
S17	32	48	19	87	0	0	0	0	0	0	0	0	0	154
S22	64	46	16	69	0	0	0	0	0	0	0	0	0	131
S23	16	1	16	21	14	2	8	38	10	71	23	19	42	265
S26	32	55	24	83	0	0	0	0	0	0	0	0	0	162
174	-	40	11	55	58	19	34	26	10	52	27	19	47	398
194	-	25	7	34	48	15	18	0	0	0	0	0	0	147
Statistics														
P value		0.011	0.017	0.038	NS	NS	NS	NS	NS	NS	NS	NS	NS	NS
95% CI		6.1-41.7	2.5-18.8	2.5-69.9										

NS; not significant.

In 4 of the 7 susceptible strains (177, 189, F44, and F65), there were no SNVs in the MDR efflux pump genes. The other 3 susceptible strains had SNVs in efflux pump genes. In the CLR-susceptible strain 179, there was a total of 350 SNVs across all four clusters of efflux pump genes. In contrast, all CLR-resistant *H. pylori* strains have SNVs in at least one of the four gene clusters. Particularly, in the *hp0605*-*hp0607* cluster, SNVs were certainly observed in all 12 resistant strains. There were significant differences in SNVs of the *hp0605*-*hp0607* cluster between susceptible and resistant strains (P < 0.05), but not in the other clusters.

Correlations between the total number of SNVs in efflux pump clusters and MICs of CLR were examined. In 10 resistant strains out of 12 isolates, MICs of CLR were ranged from 4 to 64 μg/ml and exhibited over 1 μg/ml with the criteria of CLR resistance (Table 5). We found no significant correlation between SNVs and level of CLR resistance.

To examine the common CDS variant in CLR-resistant *H. pylori*, further detailed analysis of the SNVs in the *hp0605*-*hp0607* cluster was performed (Table 6). In all CLR-resistant strains, there were no common SNVs in each *hp0605*, *hp0606*, and *hp0607* cluster. For the maximum number of common SNVs, four SNVs of *hp0605* (*hefA*) were found in 11 out of 12 resistant strains (91.6%). Of note, one CDS variant of Asn177Thr was in 11 resistant strains. In *hp0606* and *hp0607*, there were no CDS variants specific to the resistant strains, though common SNVs in 11 strains (three SNVs in *hp0606* and two SNVs in *hp0607*) were found. These results indicated that independent SNVs in the efflux pump genes may contribute to CLR susceptibility profiles for each strain.

**Table 6:** **SNVs of *hp0605-hp0607 *** **cluster in 12 CLR-resistant *H. pylori***

	**Locus tag**	**HP0605 (NP_207400.1)**							
	**Annotation**	**hypothetical protein**							
	**Coding sequence**	**c.530A > C**		**c.546A > G**		**c.804 T > C**		**c.1365 T > C**	
	**Amino acid**	**p.Asn177Thr**		**No**		**No**		**No**	
	**Nucleotide position**^**a**^	**641828**		**641844**		**642102**		**642663**	
**Strain**		**Count reads/Total reads**	**Frequency (%)**	**Count reads/Total reads**	**Frequency (%)**	**Count reads/Total reads**	**Frequency (%)**	**Count reads/Total reads**	**Frequency (%)**
S1		179/180	99.4	181/181	100	140/141	99.3	107/107	100
S2		373/373	100	392/394	99.5	319/320	99.7	295/295	100
S4		212/213	99.5	219/221	99.1	212/214	99.1	204/205	99.5
S8		507/508	99.8	530/532	99.6	478/479	99.8	503/506	99.4
S13		218/218	100	225/225	100	169/170	99.4	179/179	100
S16		343/343	100	371/371	100	325/325	100	312/313	99.7
S17		266/266	100	306/306	100	185/186	99.5	246/246	100
S22		328/328	100	348/348	100	299/299	100	287/289	99.3
S23		-		-		-		-	
S26		341/341	100	368/368	100	321/321	100	310/311	99.7
174		123/123	100	124/124	100	114/114	100	151/151	100
194		305/306	99.7	324/324	100	341/343	99.4	231/232	99.6
	**Locus tag**	**HP0606 (NP_207401.1)**							
	**Annotation**	**membrane fusion protein MtrC**							
	**Coding sequence**	**c.99 T > C**		**c.105A > G**		**c.192A > G**			
	**Amino acid**	**No**		**No**		**No**			
	**Nucleotide position**	**642841**		**642847**		**642934**			
**Strain**		**Count reads/Total reads**	**Frequency (%)**	**Count reads/Total reads**	**Frequency (%)**	**Count reads/Total reads**	**Frequency (%)**		
S1		140/140	100	129/129	100	139/140	99.3		
S2		340/340	100	324/324	100	383/385	99.5		
S4		194/194	100	192/193	99.5	221/223	99.1		
S8		496/498	99.6	472/473	99.8	505/506	99.8		
S13		203/203	100	194/194	100	-			
S16		329/331	99.4	316/317	99.7	335/335	100		
S17		233/233	100	230/230	100	272/273	99.6		
S22		295/295	100	285/285	100	320/322	99.4		
S23		109/109	100	105/105	100	110/110	100		
S26		323/325	99.4	311/312	99.7	331/331	100		
174		137/138	99.3	126/127	99.2	137/138	99.3		
194		-		-		299/301	99.3		
	**Locus tag**	**HP0607 (NP_207402.1)**							
	**Annotation**	**acriflavine resistance protein AcrB**							
	**Coding sequence**	**c.1341T>C**		**c.39A>G**					
	**Amino acid**	**No**		**No**					
	**Nucleotide position**	**644800**		**643498**					
**Strain**		**Count reads/Total reads**	**Frequency (%)**	**Count reads/Total reads**	**Frequency (%)**				
S1		159/160	99.4	123/124	99.2				
S2		345/347	99.4	280/280	100				
S4		284/286	99.3	182/182	100				
S8		585/586	99.8	431/432	99.8				
S13		227/228	99.6	164/164	100				
S16		425/425	100	295/295	100				
S17		317/317	100	198/198	100				
S22		346/347	99.7	273/275	99.3				
S23		104/104	100	106/106	100				
S26		419/419	100	295/295	100				
174		113/113	100	-					
194		-		342/344	99.4				

^a^Nucelotide position was based on NC_000915.

## Discussion

Here, we have sequenced whole genomes of *H. pylori* isolates by using the massively parallel illumina-based platform. This MiSeq platform can generate the largest amount of paired-end sequence data per sample using a chip-based bridge amplification procedure followed by sequencing by synthesis [24]. To determine the adequate total reads and average coverage per sample, six different sequencing data sets from ATCC26695 have been analyzed. We have observed that the amount of sequencing reads after trimming was directly proportional to the average coverage from 4.2- to 81.7-fold. Genome depth is a major factor that determines the ability to reconstruct genome sequences and detect genetic variants with low error rate [25]. To reach the necessary balance between the number of total reads and average coverage, over one million reads with depth of over 70-fold is sufficient for identification of genetic variants in the bacterial genome of 1.6 Mb [26,27].

However, we could not create a complete gap closure to the reference *H. pylori* genome with sufficient sequencing coverage. Illumina sequencing technique produces large amounts of short overlapping reads from the target genome [28]. The first task of analysis is to assemble these reads to larger regions of the bacterial genome. Since there is an assembly gap, we have reduced the number of false positive candidates and did not try to identify insertions or deletions (indels) of any size in the analysis. Sequencing-based indels detection methods are not well-established even utilizing paired-end data [29], and the reliable detection of indels is complex. In addition, under our approach of reference based assembly, it is difficult to detect true indels due to small assembly gaps even when sequencing the reference genome. Thus, we have selected a conservative analysis method focused on SNVs of *H. pylori* coding sequence.

In the present study, we have applied the AS-PCR method to detect mutations in the 23S rRNA gene of *H. pylori*, in which these mutations were perfectly identical in all sequencing reads at the AS-PCR assayed position. AS-PCR utilizes allele-specific primers such that the second nucleotide from the 3′ end is designed to match the site of the point mutation [30]. This AS-PCR assay is well-established to definitely identify whether a *H. pylori* strain is sensitive or resistant to CLR [31,32]. Our analysis is a successful comparison between point mutations detected by both AS-PCR and short-read sequencing. Currently, there are few studies in which genetic mutations of antimicrobial resistance identified with sequencing data are consistent with recorded variants [33,34]. In addition, next-generation sequencing could clearly distinguish a point mutation in the 23S rRNA gene in the *H. pylori* genome.

To provide future advances for treatment of *H. pylori* infections, we need to know whether variants of MDR efflux pump genes rely on drug resistance as a species-dependent intrinsic factor. The resistance-nodulation-cell division (RND) family is grouped into MDR efflux pumps in gram-negative bacteria [35]. AcrB protein, a member of the RND family, forms a homotrimer with an outer membrane protein (TolC) and a periplasmic membrane fusion protein (AcrA) [36,37]. In *H. pylori*, two TolC homologs, *hp0605* and *hp1489*, have been identified on the basis of structural similarities with outer membrane efflux protein formed RND domains [38]. In addition, three RND efflux systems, each of which consists of an accessory protein of translocase, have been identified as TolC homolog [39]. Now, four gene clusters of efflux pump systems (*hp0605*-*hp0607*, *hp0971*-*hp0969*, *hp1327*-*hp1329*, and *hp1489*-*hp1487*) are identified in *H. pylori*[23] and possibly contribute to resistance to antimicrobials, which resemble the situation in other bacteria [40]. Expression analysis in clinical isolates has shown the importance of efflux pump genes to antimicrobial agents. In response to the exposure of metronidazole, expression of TolC efflux pump gene (*hp0605*) has been shown to increase, indicating the relation with drug resistance in *H. pylori*[41]. More recently, exposure of CLR *in vitro* has induced natural transformation to the genome of strain 26695, which resulted in the point mutation of *acrB* (*hp0607*) with whole-genome sequencing [42]. To extend these previous findings, this study highlights the importance of MDR efflux pumps of *H. pylori* derived from active infections.

This study has indicated a link between specific variants of efflux pump genes and the CLR-resistant phenotype. The exact mechanism underlying these genetic mutations is unclear. Possible mechanisms of intrinsic drug resistance involve decreased drug uptake or increased drug efflux [39]. Knockout mutants for each of *hp0605* and *hp0971* have displayed drug susceptibility profiles to metronidazole, novobiocin, and sodium deoxycholate [23]. Indeed, we have found that specific mutations in *hp0605*, *hp0971*, and the other variants in efflux pump systems are significant in CLR-resistant strains. Whole-genome sequencing data include vast amounts of additional information that are currently unavailable from small scale analysis. Variants in efflux pump genes have a possible effect on the association with drug susceptibility in clinical isolates.

## Conclusions

We have provided successful validation of genotypic prediction for antimicrobial resistance with the PCR-based approach and highlighted the potential of whole-genome sequencing in the analysis of *H. pylori* isolates. These sequencing data have emphasized the significant function of the variants of efflux pump genes in CLR-resistant strains. Since we have been conservative in our analysis to focus on only well-validated SNVs in limited parts of the bacterial genome, further analyses (e.g., indels, short repeats, rearrangements, and phylogenetics) with whole genome methods, such as gap closure, could provide more detailed discrimination of the drug resistant *H. pylori* isolates.

## Methods

### *H.pylori* samples

Nineteen *H. pylori* clinical isolates were separated from gastric epithelium biopsy tissues during upper gastroduodenal endoscopy at Okinawa Prefectural Chubu Hospita, and Kobe University Hospital. All patients gave written informed consent for use of their samples for the present study, and this study was performed according to the principles of the Declaration of Helsinki. The major reference strain, ATCC26695 (NC_000915), was also used.

### *H.pylori* culture

Gastric biopsy specimens were transferred onto a Trypticase soy agar II (TSA-II)-5% sheep blood plate and cultured under microaerobic conditions (O_2_, 5%; CO_2_, 15%; N_2_, 80%) at 37°C for 5 days. One colony was picked from each primary culture plate, and seeded onto a fresh TSA-II plate. A few colonies were picked from each plate and transferred into 2 ml of brucella broth liquid culture medium containing 10% fetal calf serum and cultured for 3 days. A part of the liquid culture was stored at -80°C in 0.01 M PBS containing 20% glycerol. *H. pylori* DNA was extracted from the pellet of the liquid culture by the protease-phenol-chloroform method, suspended in 300 μl of TE buffer, and stored at 4°C until AS-PCR and sequencing.

### Antibiotic susceptibility testing

To determine the MICs of CLR, CLSI agar dilution method was used [43]. Briefly, two-fold dilutions of CLR were prepared in Muller-Hinton agar (Becton Dickinson, MD) with 5% sheep blood. Following the preparation of *H. pylori* suspension in physiological saline adjusted to a 2.0 McFarland standard, a 1 to 3 μl inoculum was spotted on agar plate. Following incubation at 37°C for 72 h under microaerophilic atomosphere, the MICs of CLR were established as the drug concentration showing no growth.

### Allele-specific PCR (AS-PCR) for 23S rRNA gene mutations

Analysis of alleles with point mutations in 23S rRNA (A2146G and A2147G) was performed according to previous reports [31,32]. The allele-specific primer sequences are listed in Additional file 4: Table S1. The PCR mixture was contained 100 ng of DNA with 10 × buffer 2.5 μl, 2 mM dNTP 2.5 μl, 25 mM MgCl_2_ 1 μl, 10 pmol primer mixtures, and 0.5 U KOD-plus DNA polymerase (TOYOBO, Osaka, Japan). Then, mixture was up to 25 μl with distilled water. The PCR condition was as follows; 94°C for 2 min and 35 cycles of 94°C for 15 sec, 60°C for 30 sec, and 68°C for 30 sec, followed by 68°C for 2 min. PCR products were determined by 4% agarose gel electrophoresis. The gels were stained with ethidium bromide to determine the product size. ATCC26695 and J99 were used as control strains for the AS-PCR.

### Preparation of genomic DNA and whole-genome sequencing

Total DNA of *H. pylori* isolated from patients and the reference ATCC26695 were sequenced. Bacterial DNA was extracted with DNeasy blood and tissue kit according to manufacturer’s guideline (Qiagen, Hilden, Germany). DNA concentration of each sample was measured with Qubit dsDNA HS assay kit (Q32851; Invitrogen, Carlsbad, CA). An <500-bp DNA library of *H. pylori* strains (50 ng or 1 ng) was prepared by using Nextera DNA Sample Prep Kit (illumina, San Diego, CA) or Nextera XT DNA Sample Prep Kit (illumina). According to the manufacturer’s instructions (version May 2012), DNA was uniformly sheared into 500-bp portions with these kits, and DNA libraries were pooled with 1% 8 pM PhiX control Spike in, and then run on MiSeq sequencer (illumina) with Reagent kit (300 cycle, paired-end). Fluorescent images were analyzed using the MiSeq Control Software, and FASTQ-formatted sequence data was created using MiSeq Reporter Analysis.

### Sequence reads mapping and SNVs detection

For used sequence data, read quality was selected at Q30 over 80% followed by the suggestion of illumina. After quality check and data trimming, Genomics Workbench 6.0.1 (CLC bio, Aarhus, Denmark) was used to assemble the sequence. The read mapping module was termed as CLC Assembly Cell 4.0, which was based on an uncompressed Suffix-Array representing the entire reference genome in a single data structure (White paper on CLC read mapper; October 10, 2012). Sequence reads were mapped against the reference genome of ATCC26695 (NC_000915), and single nucleotide variants (SNVs) were identified with probabilistic variant detection modules with default parameters and minor modifications in the mapping algorithm. Variant detection was set to 1 in Genomics Workbench 6.0.1. To exclude false-positive variants resulting from sequencing errors, we selected variants presented in > 99.0% of mapped reads with minimum coverage of 100. Insertions and deletions were also discarded. During this process, a set of high confidence variants was generated.

### Variants of MDR efflux pumps in clinical *H. pylori* strains

Generally, in gram-negative bacteria, the resistance-nodulation-cell division (RND) family is representative of MDR efflux pumps, which includes the AcrAB-TolC system in *Escherichia coli*[35]. In the *H. pylori* 26695 genome, four gene clusters of TolC homologues, *hp0605*-*hp0607* (*hefABC*), *hp0971*-*hp0969* (*hefDEF*), *hp1327*-*hp1329* (*hefGHI*), and *hp1489*-*hp1487*, have been indicated as MDR efflux pumps based on sequence similarity to other bacterial species [23] and their gene expression patterns [16].

### Statistical analysis

The associations between the genetic variants of efflux pump genes and the phenotype of CLR resistance were analyzed with Student’s t-test. Correlation coefficient between the genetic variants and MICs of CLR was also calculated, and the 95% confidence interval (CI) was used to estimate the risk. A P value of <0.05 was accepted as statistically significant. The SPSS statistical software package version 20.0 (SPSS, Inc., Chicago, IL) was used for all statistical analyses.

### Nucleotide sequence accession number

All sequence reads of 19 clinical isolates and ATCC26695 have been deposited in the DNA Data Bank of Japan Sequence Read Archive (http://www.ddbj.nig.ac.jp/index-e.html) under accession number DRA001250.

## Competing interests

The authors declared that they have no competing interests.

## Authors’ contributions

TT, AI, and RO conceived and designed the research. AI, RO, LY, and KY collected samples and performed experiments. TT, AI, and RO analyzed the data and prepared figures, interpreted results of experiments, and drafted manuscript. TT and AI edited manuscript. YM, SN, MY, and TA supervised this study. All authors read and approved the final manuscript.

## Supplementary Material

Additional file 1: Figure S1 Sequencing quality control of ATCC26695 sequence data from sample 6. ATCC26695 sequence data from sample 6 with 2,148,576 reads was subjected to the quality control analysis. The length distribution of sequencing reads **(A)**, ambiguous base-content **(B)**, and quality distribution at each base position **(C)**, are shown. Click here for file

Additional file 2: Figure S2 Flow chart of sequencing reads analysis. Click here for file

Additional file 3: Figure S3 Sequence of 23S rRNA gene at 2146 and 2147 position in CLR-susceptible F79 strain. Click here for file

Additional file 4: Table S1 Allele specific-PCR primer sequences. Click here for file
