# Supplementary figures and images for: Whole-genome sequencing of clarithromycin resistant Helicobacter pylori characterizes unidentified variants of multidrug resistant efflux pump genes
Source: Gut Pathog. 2014 Jun 26;6:27. doi: 10.1186/1757-4749-6-27 (PMC4079918; doi:10.1186/1757-4749-6-27)

Figure S1

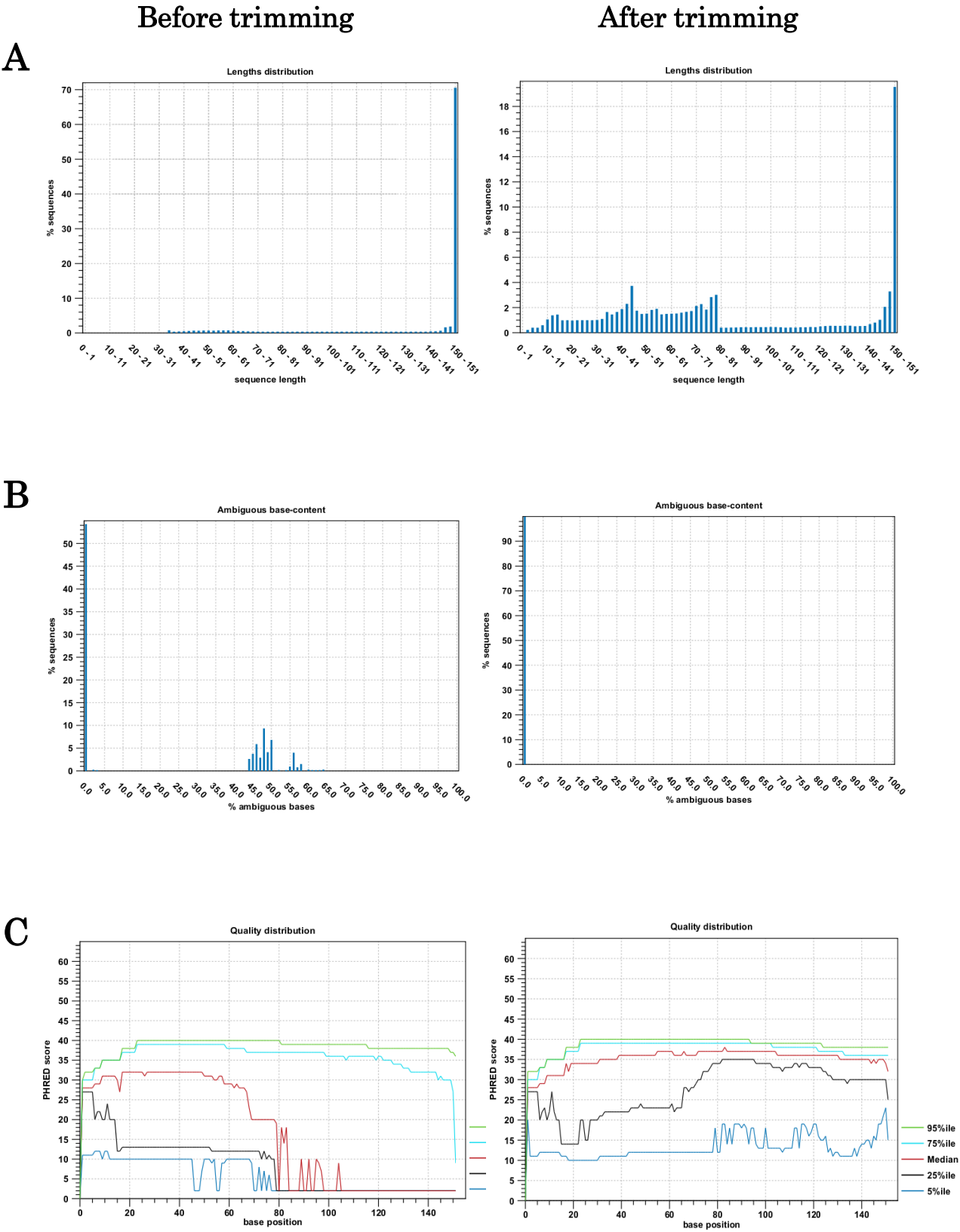

Supplement: Additional file 1: Figure S1 — Sequencing quality control of ATCC26695 sequence data from sample 6. ATCC26695 sequence data from sample 6 with 2,148,576 reads was subjected to the quality control analysis. The length distribution of sequencing reads (A), ambiguous base-content (B), and quality distribution at each base position (C), are shown. [file 1757-4749-6-27-S1.pdf]

Figure S2

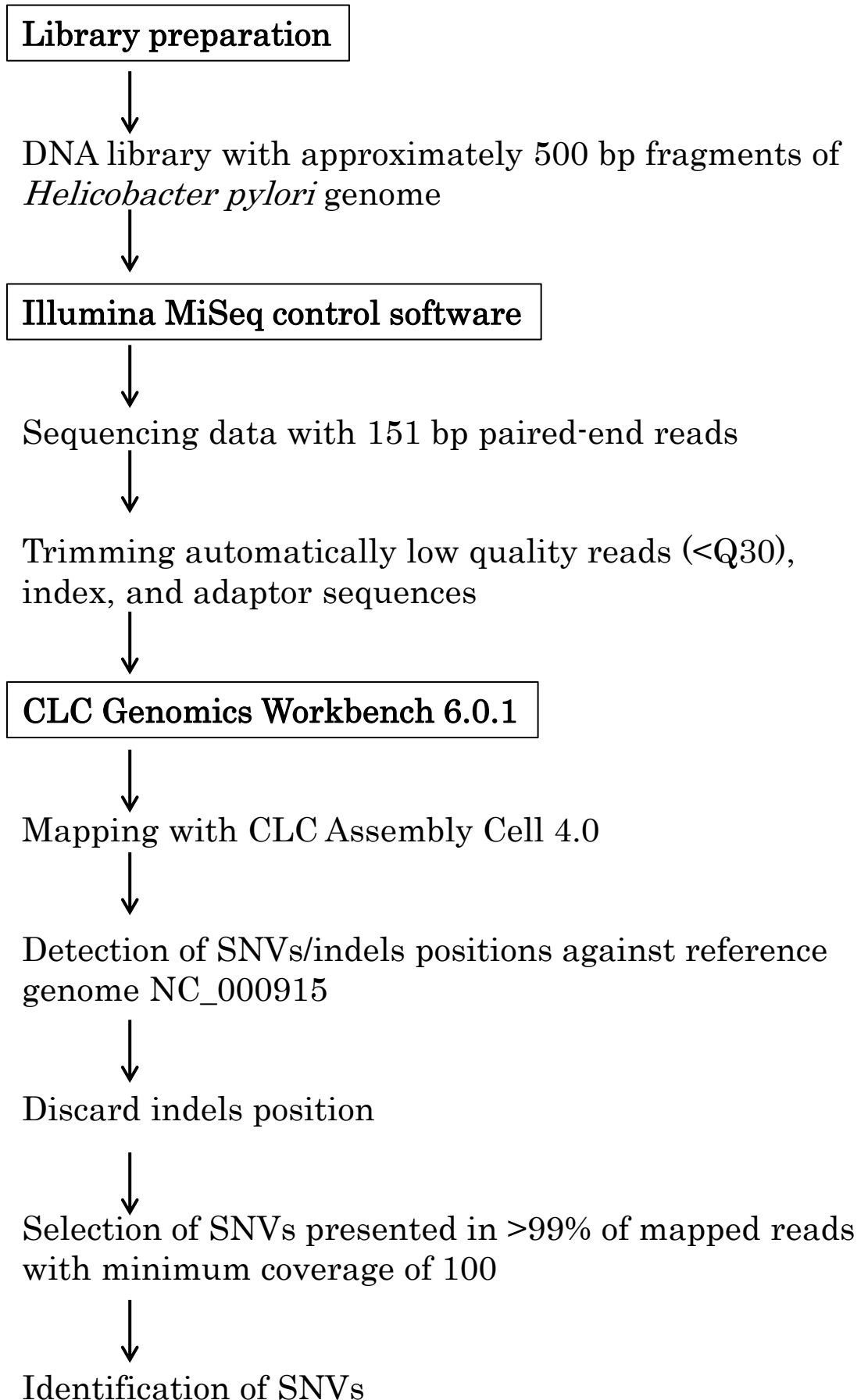

Supplement: Additional file 2: Figure S2 — Flow chart of sequencing reads analysis. [file 1757-4749-6-27-S2.pdf]

## Figure S3

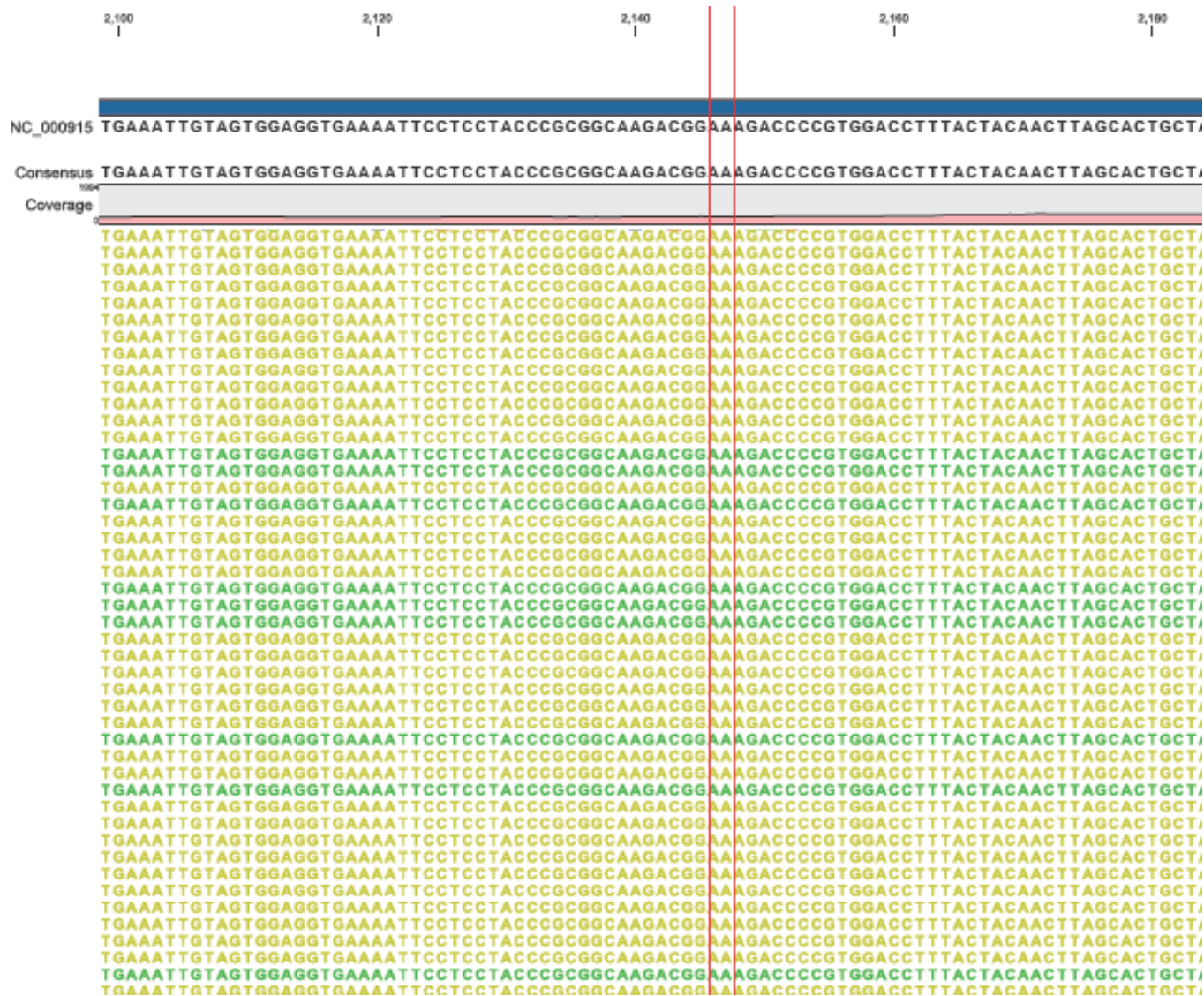

Supplement: Additional file 3: Figure S3 — Sequence of 23S rRNA gene at 2146 and 2147 position in CLR-susceptible F79 strain. [file 1757-4749-6-27-S3.pdf]
